# Supplementary material for: Can We Predict Prostate Cancer Metastasis Based on Biomarkers? Where Are We Now?
Source: Int J Mol Sci. 2023 Aug 7;24(15):12508. doi: 10.3390/ijms241512508 (PMC10420177; doi:10.3390/ijms241512508)
Supplement: Supplementary file 1 [file ijms-24-12508-s001.zip › ijms-2531188-supplementary.pdf]

Supplementary Materials

Table S1. Current commercialized tissue-based genomic tests. Modified from Sotomayor et al. [19].

Test	Company	Biomarkers	Substrate	Clinical Endpoints	Target patient	Technique	Price USD	Certification
**Decipher**	GenomDX (Vancouver, BC Canada)	22 coding and noncoding RNAs (*LASP1*, *IQGAP3*, *NFIB*, *S1PR4*, *THBS2*, *ANO7*, *PCDH7*, *MYBPC1*, *EPPK1*, *TSBP*, *PBX1*, *NUSAP1*, *ZWILCH*, *UBE2C*, *CAMK2N1*, *RABGAP1*, *PCAT-32*, *GLYATL1P4/PCAT-80*, and *TNFRSF19*	FFPE tissue from prostate tumor biopsies	Scores from 0 to 1, classifies patients as high risk 0.61 to 1.0 (1 in 5 risk of metastasis), average risk 0.46 to 0.6, or low risk 0 to 0.45 (1 in 42 risk of metastasis	Patients with localized disease on biopsy	RNA Microarray	$4,250 - $5,150.	CLIA-certified. Test is not approved by the FDA. NCCN guidelines state that the test can be considered to be used in patients with localized tumors, but studies to compare effectiveness are necessary to prove clinical utility and improve risk stratification. The test is not cited by the EAU guidelines. Limited Medicare coverage. Available 2015 in USA
**Prolaris**	Myriad Genetics, Salt Lake City, UT, USA	*31* cell cycle progression (CCP) genes*: CDKN3, RRM2, RAD54L, RAD51, CDC20, CDC2, BUB1B, PLK1, TOP2A, PTTG1, FOXM1, KIF11, KIAA0101, NUSAP1, CENPF, ASPM, DLGAP5, BIRC5, KIF20A, TK1, PBK, ASF1B, C18orf24, CDCA3, MCM10, PRC1, DTL, CEP55, CENPM, CDCA8, and ORC6L.*	1) prostate biopsy or a 2) radical prostatectomy specimen	from 0 to 10, with each 1unit increase reflecting a doubling of risk of disease progression	Patients in Active Surveillance	mRNA expression level	$3,400-$3,900	Prolaris is validated by the FDA for use in men with NCCN with low risk prostate cancer and for post-prostatectomy patients who are at high risk for prostate cancer recurrence. It has also been incorporated as an option in the NCCN and European Association of Urology guidelines for prostate cancer. Available 2012 in US
**Oncotype Dx**	Genomic Health, Redwood City, CA, USA	Use 5 reference genes (*ARF, ATP5E, CLTC, GPS1, PGK1*) and 12 genes representing 4 biologic pathways with known	paraffin-embedded prostate needle biopsy tissue	score from 0 to 100, where a value 0 is lowest risk and 100 is highest risk	Patients in Active Surveillance	real-time RT-PCR	$4,180-$5,141	CLIA-certified. $4180. Covered by Medicare . For other carriers, assistance is available from the manufacturer. Available 2013 in US

	roles in PCa tumorigenesis: the androgen pathway (*AZGP1, KLK2, SRD5A2,* and *FAM13C*), celular organization (*FLNC, GSN, TPM2,* and *GSTM2*), stromal response (*BGN, COL1A1,* and *SFRP4*) and proliferation (*TPX2*).				

Table S2. Summary of different Urologic and Oncology Societies guidelines recommendation for the use of TBGB for prediction of metastatic disease.

Society	Year of last version publication	TBGB		
		**Oncotype Dx**	**Prolaris**	**Decipher**
**American Urology Association + American Society for Radiation Oncology (AUA/ASTRO)**	2022 Localized PC, 2021 amended in 2023 Advanced PC	Not routinely recommended	Not routinely recommended	Not routinely recommended
**National Comprehensive Cancer Network (NCCN)**	2023	Initial risk stratification in low or favorable-intermediate risk	Initial risk stratification in low, intermediate and high risk.	Initial risk stratification in low, intermediate and high risk. Define adjuvant treatment post RP
**European Guidelines (EAU-EANM-ESTRO-ESUR-SIOG)**	2023	Not routinely recommended	Not routinely recommended	Describe studies about utility of post-RP but make no recommendation
**American Society of Clinical Oncology (ASCO)**	2018 Localized PC, 2020 Molecular biomarkers in localized PC, 2023 Non.castrated advanced, recurrent and mPC	Not specifically mentioned	Not specifically mentioned	Not routinely recommended
